# Techniques to Shorten a Screening Tool for Emergency Department Patients

**DOI:** 10.5811/westjem.2019.7.42938

**Published:** 2019-08-20

**Authors:** Scott G. Weiner, Jason A. Hoppe, Matthew D. Finkelman

**Affiliations:** * Brigham and Women’s Hospital, Department of Emergency Medicine, Boston, Massachusetts; † University of Colorado Denver School of Medicine, Department of Emergency Medicine, Aurora, Colorado; ‡ Tufts University School of Dental Medicine, Boston, Massachusetts

## Abstract

**Introduction:**

Screening of patients for opioid risk has been recommended prior to opioid prescribing. Opioids are prescribed frequently in the emergency department (ED) setting, but screening tools are often of significant length and therefore limited in their utility. We describe and evaluate three approaches to shortening a screening tool: creation of a short form; curtailment; and stochastic curtailment.

**Methods:**

To demonstrate the various shortening techniques, this retrospective study used data from two studies of ED patients for whom the provider was considering providing an opioid prescription and who completed the Screener and Opioid Assessment for Patients with Pain-Revised, a 24-item assessment. High-risk criteria from patients’ prescription drug monitoring program data were used as an endpoint. Using real-data simulation, we determined the sensitivity, specificity, and test length of each shortening technique.

**Results:**

We included data from 188 ED patients. The original screener had a test length of 24 questions, a sensitivity of 44% and a specificity of 76%. The 12-question short form had a sensitivity of 41% and specificity of 75%. Curtailment and stochastic curtailment reduced the question length (mean test length ranging from 8.1–19.7 questions) with no reduction in sensitivity or specificity.

**Conclusion:**

In an ED population completing computer-based screening, the techniques of curtailment and stochastic curtailment markedly reduced the screening tool’s length but had no effect on test characteristics. These techniques can be applied to improve efficiency of screening patients in the busy ED environment without sacrificing sensitivity or specificity.

## INTRODUCTION

Screening tools have been developed for emergency department (ED) patients to help detect multiple diseases and risk factors, ranging from nutrition status to sepsis to suicide risk.^1^–^3^ These tools vary in length and the time needed to complete them, and utilization is likely impacted by competing interests, priorities and ease of use. In the busy ED environment, brevity – while maintaining accuracy – is of the essence.

The United States is in the midst of an opioid crisis: an average of 134 people per day died due to an opioid-related overdose in 2017.^4^ The crisis was declared a national public health emergency in 2017.^5^ The ED is at the epicenter of the opioid crisis, for its role in prescribing opioids for acute pain, treating medical complications of injection drug use, and treating opioid use disorder. It is an area where screening for opioid risk could potentially be impactful.^6^ Although emergency physicians provide a relatively small amount of opioids compared with other specialties,^7^,^8^ there is evidence that the first opioid prescription given in the ED can portend long-term opioid use.^9^,^10^ Therefore, screening for opioid-related risk prior to a new prescription from the ED would be prudent and is also in alignment with multiple guidelines, including those by the Centers for Disease Control and Prevention and multiple cities and states.^11^–13

Unfortunately, the exact definition of what it means to “screen” a patient is unclear. Available screening tools vary in length but typically require many questions. For example, the full-length Drug Abuse Screening Test has 20 questions. The Opioid Risk Tool is 10 questions in length, but each answer is associated with a different point value and is also different for females vs males.^14^ The Screener and Opioid Assessment for Patients with Pain-Revised (SOAPP-R), perhaps the most rigorously studied and validated screening tool for opioid-related risk in the ED setting and elsewhere, is 24 questions in length,^15^–^16^ quite long for a tool that could be administered to every ED patient receiving an opioid prescription. Previous work has evaluated administering the SOAPP-R on a tablet computer as a means to allow patients to complete the screener and have their results tallied without additional ED staff time required.^17^ But for this and other screening tools used in the ED environment, discovering a way to shorten the actual number of questions required may make the screening tools more desirable for implementation.

The purpose of this study was to describe and explore three ways to reduce the length of a screening tool. Using SOAPP-R as an example, we studied shortened forms as well as techniques called curtailment and stochastic curtailment, which we define in detail below. The ultimate goal was to shorten the screening tool while not losing predictive value. Our secondary aim was to inform the reader about these techniques, which may be applied to other screening tools as well.

## METHODS

The study is a retrospective evaluation of SOAPP-R results from two prospectively enrolled convenience samples of ED patients for whom the emergency provider was considering prescribing an opioid to treat pain. The first cohort included 82 adult patients presenting to an urban, academic trauma center in Massachusetts with approximately 42,000 annual visits between May–August 2013. The second cohort included 106 adult patients presenting to an urban, academic trauma center in Colorado with approximately 100,000 annual visits between June–August 2016. The study was approved by the institutional review boards at both institutions. We did not calculate an a priori sample size as this was an analysis of preexisting data and the purpose of this paper was to demonstrate various shortening techniques.

The methodology and results have been described elsewhere in depth.^17^,^18^ Briefly, to be eligible for enrollment, patients had to have had an acute, painful condition for which the treating emergency physician was considering treating with an opioid analgesic. Patients completed SOAPP-R on a tablet computer; they were informed that the results would not be shared with their treating physician. The tablet computer recorded patients’ answers on the screening tool. The physician also accessed each patient’s prescription drug monitoring program (PDMP) record, and a trained research assistant recorded the number of Drug Enforcement Administration schedule II–V medications, subset number of opioids, number of prescribers used for all schedule II–V medications, and number of pharmacies used to fill these medications in the previous 12 months. For the purpose of the study, we defined a high-risk prescription history as having ≥4 opioid prescriptions and ≥4 providers for schedule II–V medications in the previous 12 months, as has been used in prior research.^19^,^20^

**Population Health Research Capsule**

What do we already know about this issue?

*Several opioid screening tools have been implemented in the ED setting, but their acceptance has been limited, likely due to their length*.

What was the research question?

*Can shortening techniques such as curtailment and stochastic curtailment be applied to an opioid screening tool*?

What was the major finding of the study?

*An opioid screening tool can be shortened considerably without losing predictive power*.

How does this improve population health?

*Techniques such as short forms, curtailment, and stochastic curtailment can be used to shorten screening tools, which may increase their use in the ED setting*.

For this study, we applied three techniques to shorten the full-length screening tool: short form; curtailment; and stochastic curtailment (SC). Creation of the 12-question short form has been described previously.^21^ In sum, LASSO (least absolute shrinkage and selection operator) logistic regression was used to determine which questions to include. The 12-question version had screening characteristics similar to the full-length version and the highest acceptance by an expert panel.^21^ Although initially a cutoff score of 10 or greater was suggested, further work determined that a score of 9 or greater indicating high risk produced the best test characteristics.^22^ This short form can be administered on paper, similar to the original SOAPP-R.

As opposed to the fixed-length short form, curtailment is a variable-length testing method. With curtailment, a computer (such as a tablet or smartphone) analyzes each response as it is entered and determines a) whether the number of points on the screening tool meets criteria for the respondent to be at risk, or b) whether the respondent could not achieve a number of points sufficient to be at risk with the number of questions remaining. As an example, the SOAPP-R contains 24 questions with a possibility of 0–4 points for each question. Having a score of 18 or higher indicates “high risk.” Once a respondent has 18 points, the screener ends as they are already determined to be high risk. Conversely, if the respondent has a cumulative score of no greater than 13 after the first 23 items, it would be impossible to be high risk even if they received four points for the final question; so it would end after the 23rd question. With this methodology, the number of questions varies for each individual, depending on how they respond to questions.

Stochastic curtailment is another stopping rule that halts testing not only at the same time that curtailment does, but in other specific circumstances as well. Specifically, SC also stops early when there is either a high probability that the full-length questionnaire will provide a high-risk classification (in which case stochastic curtailment makes an immediate classification of high risk), or a high probability that the full-length questionnaire will provide a low-risk classification (in which case SC makes an immediate classification of low risk). A typical cut-off would be a 95% probability, so that if a subject has a 95% or greater chance of being high risk based on previous answers, the screening tool would end. Again, the number of questions would vary for each participant but the length would be shorter than simple curtailment in most scenarios. Previous research on other screening tools (CES-D, COMM and Medicare Health Outcomes Survey) determined that the number of questions can be decreased by over 50% while having the same predictive outcome as the original screening tools at least 97% of the time.^23^–^25^ For this study, we evaluated probabilities of 95% (SC-95) and 99% (SC-99). Data analysis was performed with R (www.r-project.org).

## RESULTS

From the original studies, the following test characteristics were determined. In the first cohort, 93 patients were approached and 82 patients (88.2%) completed the study and had complete data. The mean score on SOAPP-R was 16.0 (standard deviation [SD] 12.8). Twenty-seven patients (32.9%) had a score ≥18. The test characteristics of SOAPP-R to detect high-risk prescription history were sensitivity 54% and specificity 71%. In the second cohort, 154 patients were approached and 106 patients (68.8%) completed the study and had complete data. The mean score on SOAPP-R was 12.8 (SD 10.3). Twenty-five patients (23.6%) had a score ≥18. The test characteristics of SOAPP-R to detect high-risk prescription history in this cohort were sensitivity 38% and specificity 80%. Combining the two cohorts (n=188), the sensitivity was 44% and the specificity was 76%.

The test characteristics for the full-length SOAPP-R, a shortened 12-question SOAPP-R with cutoff score of ≥9, curtailment, and stochastic curtailment (SC-95 and SC-99) are demonstrated in Table 1. The short form reduced the number of questions from 24 to 12 at the expense of a slightly decreased sensitivity (44% to 41% in the combined cohort). Curtailment and both techniques of stochastic curtailment produced nearly identical test characteristics as the original SOAPP-R, but with markedly decreased numbers of questions (from 24 questions to a mean of 19.7 for curtailment, 11.8 for SC-99 and 8.1 for SC-95) in the combined cohort.

## DISCUSSION

In our study, we have demonstrated that it is possible to shorten an opioid-risk screening tool for ED patients. Versions using curtailment and stochastic curtailment would have shortened the number of questions for the vast majority of patients. Furthermore, the diagnostic accuracy of these tests was about the same as the original screener in every permutation. Indeed, in the combined cohort, the 95% probability SC had a mean test length of 8.1 (compared to 24 questions for the full screener), and essentially unchanged sensitivity and specificity. Only the fixed-length, 12-question short form had a slightly decreased sensitivity, which is likely to be irrelevant in clinical practice.

The sensitivity and specificity when using curtailment are just as high as those of the full-length screener because the technique tracks the respondent’s answers and only stops early when the classification of the full-length screener has been determined with certainty, making the exact same classification that the full-length screener would make. Similarly, stochastic curtailment only stops early when the classification of the full-length screener has been determined to a high level of probability. For all of these versions, the sensitivity was low and the specificity was higher. Therefore, in this clinical situation each version of the SOAPP-R exhibited greater success in identifying low-risk patients than in identifying high-risk patients.

The practical limitation with curtailment and stochastic curtailment is that they require the use of a computer to administer. Our previous work demonstrated that ED patients can use a tablet computer to perform screening and that they have little difficulty and high satisfaction using the tablet for this purpose.^17^ Still, there are several downsides to be considered, such as the need to safely store, charge and clean the tablet between patient use, as well as the possibility of theft and the added expense of purchasing a device.

There are other options with potential applicability to the ED setting. It is possible to reduce the reduce the length of the SOAPP-R to a uniform 12 questions, as previously described, or even down to eight questions.^21^, ^26^ Regarding these short forms, which do not require a computer to administer, their sensitivity and specificity would be expected to be similar to those of the full-length screener because the short forms were developed specifically to retain the items most predictive of the outcome. In developing the 12-item test, questions from the original SOAPP-R asking about aberrant use of pain medication, such as how often the medication ran out early or how often the individual used more pain medication than they were supposed to, were the most predictive of the outcome. Conversely, the questions asking patients if they felt bored or had any close friends with an alcohol or drug problem were the least predictive. Notably, the technique of curtailment does not itself provide an indication of which items are most (and least) predictive of the outcome, nor does it allow us to determine the number of items to be administered in advance. Taking the process a step further, it is then possible to administer the shorter static forms on a computer and apply curtailment techniques, reducing the number of questions even more.^27^ All of this work supports the concept that lengthy screening tools that have been developed for non-ED settings can potentially be repurposed and made more efficient for the frenetic and time-sensitive environment of the ED without a negative effect on the predictive value of the screening tool.

It should be noted that this study serves to demonstrate the concept of shortening the SOAPP-R but does not yet provide compelling evidence that this particular screening tool should be used in the ED setting. A recent systematic review comparing SOAPP-R and other commonly used opioid-screening tools found that the validity and reliability of all of the screeners they investigated were lacking and could not be validated for use in the ED setting.^28^ Our studies of the SOAPP-R, for example, are based on patients with four or more providers for four or more opioid prescriptions in the prior 12 months. That cutoff was chosen empirically as a higher risk quality, but has not yet been adequately tied to a concrete clinical outcome such as overdose death. Furthermore, another study discovered that about two-thirds of patients who presented to an ED with opioid dependence had no prescriptions documented in their state PDMP, indicating that it is an imperfect outcome measure.^29^

Recently, there has been work to shorten other screening tools for ED use. For example, a study evaluating the Beck Scale for Suicide Ideation was amenable to computer adaptive testing, in which the next question administered was dependent on the patient’s answer to the previous questions.^30^ Similar findings had been previously described in non-ED patients as well.^31^ With this methodology, the 19-question score could be reduced to just four questions in both studies. Future work like this, in which questions are asked in a non-linear fashion and the screener is ended when there is significant probability of detecting a result shows great promise for future computer-based screening.

## LIMITATIONS

The study is subject to the same limitations as the source studies, including that patients were enrolled in a convenience sample fashion, non-English speaking patients were excluded, and that the “gold standard” outcome measure of four or more opioid prescriptions and four or more prescribers for controlled substances in 12 months was imperfect. However, the primary goal of the paper was to demonstrate the applicability of various shortening techniques on a tool that could be used in the ED environment. This was a real-data simulation study that may produce different results than a prospectively collected sample. As an example, test results were determined post hoc based on subjects’ responses on the full length SOAPP-R. With the short form, certain questions are eliminated and context effects – how a preceding question affects how a respondent answers a subsequent question – may cause variation not detectable by our methods.

## CONCLUSIONS

In an ED population completing computer-based screening, the techniques of short forms, curtailment and stochastic curtailment markedly reduced the screening tool’s length but had negligible effects on test characteristics. These techniques can be applied to improve the efficiency of screening tools used in the busy ED environment.

## Figures and Tables

**Table:** 

Cohort 1 (n=82)					

	Sensitivity	Specificity	Mean Number of Questions	SD of Test Length	% of Tests Shortened
Full-length SOAPP-R	0.54	0.71	24.0	0.0	0.0
Shortened SOAPP-R	0.46	0.71	12.0	0.0	100.0
Curtailment	0.54	0.71	19.1	5.3	85.4
SC-99	0.54	0.71	12.3	6.4	87.8
SC-95	0.54	0.74	8.2	6.2	95.1

*SD*, standard deviation; *SOAPP-R*, Screener and Opioid Assessment for Patients with Pain-Revised; *SC*, stochastic curtailment.

**Table:** 

Cohort 2 (n=106)					

	Sensitivity	Specificity	Mean Number of Questions	SD of Test Length	% of Tests Shortened
Full-length SOAPP-R	0.38	0.80	24.0	0.0	0.0
Shortened SOAPP-R	0.38	0.79	12.0	0.0	100.0
Curtailment	0.38	0.80	20.2	4.4	88.7
SC-99	0.38	0.80	11.4	6.0	95.3
SC-95	0.38	0.80	8.0	6.1	100.0

*SD*, standard deviation; *SOAPP-R*, Screener and Opioid Assessment for Patients with Pain-Revised; *SC*, stochastic curtailment.

**Table:** 

Cohort Combined (n=188)					

	Sensitivity	Specificity	Mean Number of Questions	SD of Test Length	% of Tests Shortened
Full-length SOAPP-R	0.44	0.76	24.0	0.0	0.0
Shortened SOAPP-R	0.41	0.75	12.0	0.0	100.0
Curtailment	0.44	0.76	19.7	4.8	87.2
SC-99	0.44	0.76	11.8	6.2	92.0
SC-95	0.44	0.77	8.1	6.1	97.9

*SD*, standard deviation; *SOAPP-R*, Screener and Opioid Assessment for Patients with Pain-Revised; *SC*, stochastic curtailment.

## References

[1] Raupp D, Silva FM, Marcadenti A Nutrition screening in public hospital emergency rooms: Malnutrition Universal Screening Tool and Nutritional Risk Screening-2002 can be applied. Public Health.

[2] Filbin MR, Thorsen JE, Lynch J (2018). Challenges and opportunities for emergency department sepsis screening at triage. Sci Rep.

[3] Mullinax S, Chalmers CE, Brennan J (2018). Suicide screening scales may not adequately predict disposition of suicidal patients from the emergency department. Am J Emerg Med.

[4] National Institute on Drug Abuse (2019). Overdose Death Rates.

[5] The White House Ending America’s opioid Ccrisis.

[6] Hawk K, D’Onofrio G (2018). Emergency department screening and interventions for substance use disorders. Addict Sci Clin Pract.

[7] Weiner SG, Baker O, Rodgers AF (2018). Opioid prescriptions by specialty in Ohio, 2010–2014. Pain Med.

[8] Axeen S, Seabury SA, Menchine M (2018). Emergency department contribution to the prescription opioid epidemic. Ann Emerg Med.

[9] Shah A, Hayes CJ, Martin BC (2017). Characteristics of Initial prescription episodes and likelihood of long-term opioid use - United States, 2006–2015. MMWR Morb Mortal Wkly Rep.

[10] Hoppe JA, Kim H, Heard K (2015). Association of emergency department opioid initiation with recurrent opioid use. Ann Emerg Med.

[13] Weiner SG, Baker O, Poon SJ, Rodgers AF, Garner C, Nelson LS, Schuur JD (2017). The effect of opioid prescribing guidelines on prescriptions by emergency physicians in Ohio. Ann Emerg Med.

[14] Webster LR, Webster RM (2005). Predicting aberrant behaviors in opioid-treated patients: preliminary validation of the Opioid Risk Tool. Pain Med.

[15] Butler SF, Fernandez K, Benoit C (2008). Validation of the revised Screener and Opioid Assessment for Patients with Pain (SOAPP-R). J Pain.

[16] Butler SF, Budman SH, Fernandez KC (2009). Cross-validation of a screener to predict opioid misuse in chronic pain patients (SOAPP-R). J Addict Med.

[17] Weiner SG, Horton LC, Green TC (2015). Feasibility of tablet computer screening for opioid abuse in the emergency department. West J Emerg Med.

[18] Kim K, Hoppe JA, Kiemele ER (2017). A comparison of three screening tools for aberrant opioid drug-related behavior in the emergency department. Acad Emerg Med.

[19] Weiner SG, Griggs CA, Mitchell PM (2013). Clinician impression versus prescription drug monitoring program criteria in the assessment of drug-seeking behavior in the emergency department. Ann Emerg Med.

[20] Weiner SG, Horton LC, Green TC (2016). A comparison of an opioid abuse screening tool and prescription drug monitoring data in the emergency department. Drug Alcohol Depend.

[21] Finkelman MD, Smits N, Kulich RJ (2017). Development of short-form versions of the Screener and Opioid Assessment for Patients with Pain-Revised (SOAPP-R): A Proof-of-Principle Study. Pain Med.

[22] Finkelman MD, Jamison RN, Kulich RJ (2017). Cross-validation of short forms of the Screener and Opioid Assessment for Patients with Pain-Revised (SOAPP-R). Drug Alcohol Depend.

[23] Smits N, Finkelman MD, Kelderman H (2016). Stochastic curtailment of questionnaires for three level classification: shortening the CES-D for assessing low, moderate, and high risk of depression. Appl Psychol Meas.

[24] Finkelman MD, Kulich RJ, Zoukhri D (2013). Shortening the Current Opioid Misuse Measure via computer-based testing: a retrospective proof-of-concept study. BMC Med Res Methodol.

[25] Finkelman MD, He Y, Kim W (2011). Stochastic curtailment of health questionnaires: a method to reduce respondent burden. Stat Med.

[26] Black RA, McCaffrey SA, Villapiano AJ, Jamison RN, Butler SF (2018). Development and validation of an eight-item brief form of the SOAPP-R (SOAPP-8). Pain Med.

[27] Finkelman MD, Jamison RN, Magnuson B (2019). Computer-based testing and the 12-item Screener and Opioid Assessment for Patients with Pain-Revised: a combined approach to improving efficiency. J Appl Behav Res.

[28] Sahota PK, Shastry S, Mukamel DB (2018). Screening emergency department patients for opioid drug use: a qualitative systematic review. Addict Behav.

[29] Hawk K, D’Onofrio G, Fiellin DA (2018). Past-year prescription drug monitoring program opioid prescriptions and self-reported opioid use in an emergency department population with opioid use disorder. Acad Emerg Med.

[30] Boudreaux ED, De Beurs DP, Nguyen TH (2018). Applying computer adaptive testing methods to suicide risk screening in the emergency department. Suicide Life Threat Behav.

[31] De Beurs DP, de Vries AL, de Groot MH (2014). Applying computer adaptive testing to optimize online assessment of suicidal behavior: a simulation study. J Med Internet Res.

